# Pilot Study of a Powered Exoskeleton for Upper Limb Rehabilitation Based on the Wheelchair

**DOI:** 10.1155/2019/9627438

**Published:** 2019-12-17

**Authors:** Qiaoling Meng, Qiaolian Xie, Haicun Shao, Wujing Cao, Feng Wang, Lulu Wang, Hongliu Yu, Sujiao Li

**Affiliations:** ^1^Institute of Rehabilitation Engineering and Technology, University of Shanghai for Science and Technology, Shanghai, China; ^2^Shanghai Engineering Research Center of Assistive Devices, Shanghai, China; ^3^Key Laboratory of Neural-functional Information and Rehabilitation Engineering of the Ministry of Civil Affairs, Shanghai, China

## Abstract

To help hemiplegic patients with stroke to restore impaired or lost upper extremity functionalities efficiently, the design of upper limb rehabilitation robotics which can substitute human practice becomes more important. The aim of this work is to propose a powered exoskeleton for upper limb rehabilitation based on a wheelchair in order to increase the frequency of training and reduce the preparing time per training. This paper firstly analyzes the range of motion (ROM) of the flexion/extension, adduction/abduction, and internal/external of the shoulder joint, the flexion/extension of the elbow joint, the pronation/supination of the forearm, the flexion/extension and ulnar/radial of the wrist joint by measuring the normal people who are sitting on a wheelchair. Then, a six-degree-of-freedom exoskeleton based on a wheelchair is designed according to the defined range of motion. The kinematics model and workspace are analyzed to understand the position of the exoskeleton. In the end, the test of ROM of each joint has been done. The maximum error of measured and desired shoulder flexion and extension joint angle is 14.98%. The maximum error of measured and desired elbow flexion and extension joint angle is 14.56%. It is acceptable for rehabilitation training. Meanwhile, the movement of drinking water can be realized in accordance with the range of motion. It demonstrates that the proposed upper limb exoskeleton can also assist people with upper limb disorder to deal with activities of daily living. The feasibility of the proposed powered exoskeleton for upper limb rehabilitation training and function compensating based on a wheelchair is proved.

## 1. Introduction

Upper extremity motor function disorder is one of the most common rehabilitation problems of hemiplegic patients with stroke [1]. The upper extremity motor function plays a key role in self-care and social activities. The upper extremity motor function disorder significantly lowers the life quality of hemiplegic patients with stroke [2, 3]. Due to the complex structure and functional requirement of the upper limb, the rehabilitation process of the impaired upper extremity functionality is a long and slow process. Because of the specificity of hemiplegic patients in diagnosis, treatment, and rehabilitation, it brings a series of severe psychological and financial stress for patients [4]. The outcome of upper limb motor rehabilitation depends on duration, intensity and task orientation of the training. The therapists assisting patients have to bear a significant burden. As a result, the duration of primary upper limb rehabilitation is becoming shorter [5]. To deal with these problems, robotic rehabilitation devices with the ability to conduct repetitive tasks and provide assistive force have been proposed.

The upper limb rehabilitation robots can be divided into two types according to the service environment. One is mainly used in the hospital and shared by several patients. The upper limb rehabilitation robots used in the hospital are often designed for rehabilitation training and difficult to move. Loris et al. introduced a dual exoskeleton robot called automatic recovery arm motility integrated system. The system was developed to enable therapists to define and apply patient-specific rehabilitation exercises with multidisciplinary support by neurologist, engineers, ICT specialists and designers [6]. Farshid et al. presented the GENTLE/S system for upper limb rehabilitation. The system comprised a 3-degree-of-freedom (DOF) robot manipulator with an extra 3 DOFs passive gimbal mechanism, an exercise table, computer screen, overhead frame, and chair [7]. Dongjin Lee et al. proposed a clinically relevant upper-limb exoskeleton that met the clinical requirements. The pilot test showed that the safety for robot-aided passive training of patients with spasticity could be guaranteed [8]. The other is mainly used in the home to assist a single patient in activities of daily living. A lightweight and ergonomic upper-limb rehabilitation exoskeleton named CLEVER ARM was proposed by Zeiaee et al. The wearable upper limb exoskeleton was to provide automated therapy to stroke patients [9]. Feiyun et al. presented a seven DOFs cable-driven upper limb exoskeleton for post-stroke patients. The experimental results showed that the activation levels of corresponding muscles were reduced by using the 7 DOFs cable-driven upper limb exoskeleton in the course of rehabilitation [10]. In fact, the main function of upper extremity rehabilitation devices is to provide the physical training and assist the patients with hemiplegia to perform the activities of daily living. However, hospital or home used rehabilitation robot research has just focused on one respect. Indeed, the research on the upper extremity rehabilitation devices would focus on both aspects of assisting and training. Therefore, it is important for the design of upper limb rehabilitation robot to combine the rehabilitation training and assisting function.

The stationary upper extremity rehabilitation robot cannot solve the movability problem and perform the activities of daily living (ADL). The wearable exoskeleton devices are limited by the weight. In addition, whether the range of motion is in line with the physiological joints directly determines the rehabilitation effect. Therefore, the key questions can be summarized as follows. Can we transform the weight of the upper limb exoskeleton to another movable device instead of wearing by patients? How to guarantee the design of upper limb exoskeleton joint axis in line with the human joint movement axis?

To deal with the above questions, some researchers have made useful explorations. Kiguchi et al. proposed a mechanism and control method of a mobile exoskeleton robot based on a wheelchair for 3 DOFs upper-limb motion assist [11]. The first problem of transforming weight can be solved by design based on a wheelchair. The physical rehabilitation training can be realized on a wheelchair instead of a stationary place. The ADL can be assisted by the powered upper limb exoskeleton on a moving platform. However, the rotation axis of each joint (shoulder joint and elbow joint) is moving with the movement of the upper limb. The gap between the exoskeleton and human arm is also changing by following their movement. It does not consider the problem about the movement consistency of the exoskeleton joint rotation axis and the human joint. As for this problem, Vitiello et al. proposed an elbow exoskeleton with double-shelled links to allow an ergonomic physical human-robot interface and a four-degree-of-freedom passive mechanism to allow the user's elbow and robot axes to be constantly aligned during movement [12]. However, it focused on the elbow. The whole upper limb rehabilitation was not considered. In this work, we present a novel solution for the two mentioned problems. The range of motion of the upper extremity exoskeleton based on a wheelchair is defined through the normal people test. The 6 DOFs exoskeleton based on a wheelchair is designed according to the defined range of motion. The pursuit movement experiment and the assistive movement of drinking water of the prototype are done to verify the feasibility of the design.

## 2. Materials and Methods

### 2.1. Definition of ROM of Each Joint for the Specific Upper Limb Exoskeleton on a Wheelchair

To ensure the safety of using an upper limb exoskeleton on a wheelchair, it is necessary to know the ROM of the human upper limb on the wheelchair.

The parts of the upper limb taken into account in the design of an exoskeleton are shoulder, arm, elbow, wrist, and hand. Hand is excluded in an entire upper extremity exoskeleton design because of its complexity and dexterous characteristic. Therefore, this work only analyzes the ROM of the shoulder joint, elbow joint, and wrist joint. And then the upper limb exoskeleton designed in this paper must conform to the ROM of these joints.

#### 2.1.1. Apparatus

**Figure 1:**
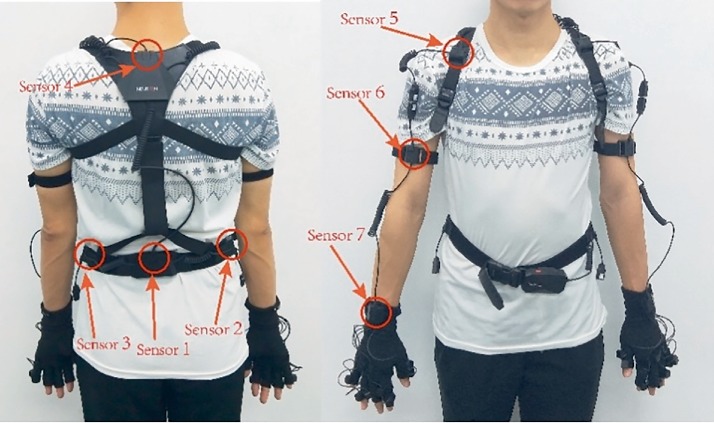
Experimental map of wearing sensor device.

The apparatus consists of a wheelchair and a motion analysis system. The motion analysis system can transmit data in real time. It was made in JIANGSU NEUCOGNIC MEDICAL CO., LTD. The system can measure the ROM of the shoulder joint, elbow joint and wrist joint of a person who sits on a common wheelchair. In Figure 1, there are two inertial sensors located at the upside and downside of backbone, and ten inertial sensors located at the upper limb (shoulder, upper arm, forearm, palm, and hand), respectively. All of the sensors in this system can measure the angles in *x*-, *y*- and *z*-axis. Sensor 1 and Sensor 4 are utilized to measure the ROM of the rear waist as the referring data. Sensor 4 and Sensor 6 are utilized to measure the ROM of the shoulder joint as the referring data. Sensor 6 and Sensor 7 are utilized to measure the ROM of the elbow joint as the referring data. Sensor 7 and hand sensor are utilized to measure the ROM of wrist joint as the referring data.

#### 2.1.2. Participants

A pilot test was to obtain the ROM of human upper limb on a wheelchair, a tester wore sensor device and sat on a wheelchair. The details of the tester are shown in Table 1. It is worth noting that rehabilitation devices are often personalized. Therefore, this work aims to provide a study approach instead of obtaining several testing data by choosing many testers.

#### 2.1.3. Measuring ROM of Human Upper Limb on a Wheelchair

The shoulder, elbow, forearm and wrist joints have 7 DOFs, consisting of shoulder's flexion/extension, adduction/abduction, internal rotation/external rotation, elbow's flexion/extension and forearm supination/pronation, wrist's flexion/extension and ulnar/radial deviation. It requires seven test movements to measure the ROM of upper limb joints.

**Figure 2:**
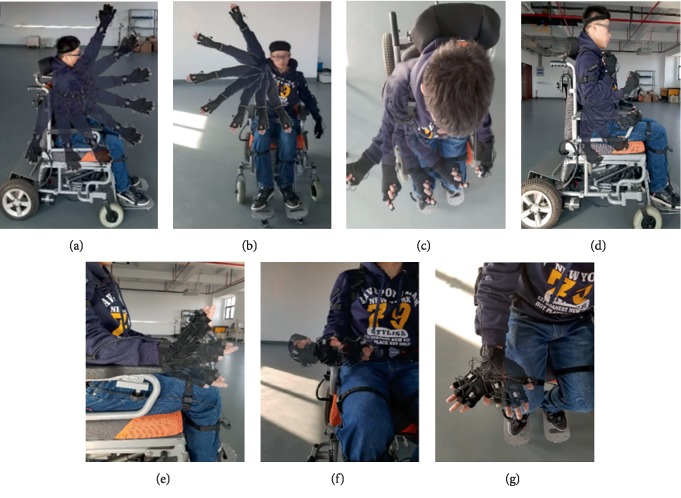
The procedure of measuring experiment. (a) Flexion and extension of the shoulder joint. (b) Adduction and abduction of the shoulder joint. (c) Internal rotation and external rotation of the shoulder joint. (d) Flexion and extension of the elbow joint. (e) Supination and pronation of the forearm. (f) Flexion and extension of the wrist joint. (g) Ulnar deviation and radial deviation.

The shoulder joint was flexion/extension, adduction/abduction, internal rotation/external rotation performed respectively and joint angles were collected in real time. We defined that the cycle of motion was between the attainable maximum ROM of the corresponding DOFs, for instance, the flexion and the extension. Each couple motion had been done for 50 cycles. The accelerate and decelerate cycles were not included in the 50 cycles due to instability problems. The procedure is shown in Figure 2.

#### 2.1.4. Data Analysis

The aim of this paper is to bring suitable and safety training to the patients. Therefore, the ROM of upper limb joints should be reasonable. We used the interval estimation to insure the measured data were reliable. We defined the maximum ROM of each degree of freedom as the parameter to be estimated. They are the maximum ROMs of flexion of the shoulder joint, the extension of the shoulder joint, the adduction of the shoulder joint, the abduction of the shoulder joint, the internal of the shoulder joint, the external of the shoulder joint, the flexion of the elbow joint, the extension of the elbow joint, the pronation of the forearm, the supination of the forearm, the flexion of the wrist joint, the extension of the wrist joint, the ulnar of the wrist joint, and the radial of the wrist joint, which are described as ^*S*^*θ*_max_^flex^, ^*S*^*θ*_max_^*ext*n^, ^*S*^*θ*_max_^add^, ^*S*^*θ*_max_^abd^, ^*S*^*θ*_max_^int^, ^*S*^*θ*_max_^*ext*l^, ^E^*θ*_max_^flex^, ^E^*θ*_max_^*ext*n^, ^F^*θ*_max_^pron^, ^F^*θ*_max_^supi^, ^W^*θ*_max_^flex^, ^W^*θ*_max_^extn^, ^W^*θ*_max_^ulnar^, and ^W^*θ*_max_^radical^, respectively. In this work, we chose 25 samples for each parameter and applied the Wald method for censored data (*α* ≤ 0.05). The confidence intervals of the average value for each parameters were calculated and shown in Table 2. According to the safety principles and the confidence, the interval offline were chosen as the desired value of each DOF. It is worth noting that the accuracy of ROM is no more than 5° in rehabilitation training. Therefore, the values of ^*S*^*θ*_max_^*ext*l^, ^F^*θ*_max_^pron^, and ^W^*θ*_max_^ulnar^ were defined to less than the confidence interval.

However, in the actual design of an upper limb exoskeleton, multiple DOFs will increase the complexity. In the measuring test, we found that the flexion/extension of the shoulder joint, the adduction/abduction of the shoulder joint, and the flexion/extension of the elbow joint are the critical DOFs in most of movements of the upper limb. Also, the three DOFs of the upper limb were often used in ADL and could complete most movements. Therefore, the three DOFs were chosen as active DOFs in the work.

### 2.2. Mechanical Design

#### 2.2.1. Description of Exoskeleton Mechanical Design with 6 DOFs

**Figure 3:**
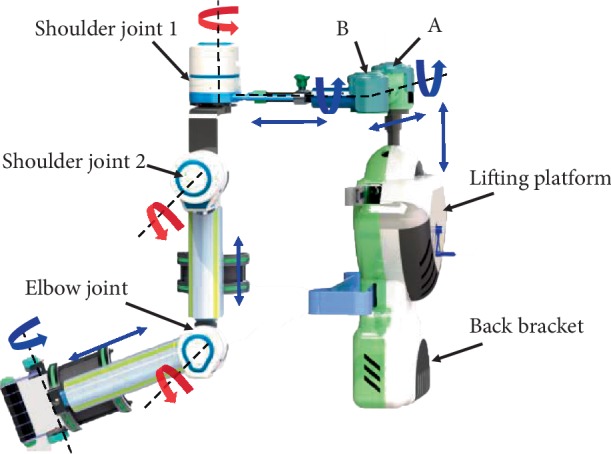
The overall structure of the upper limb rehabilitation robot based on the wheelchair platform.

Based on the above measurement and analysis of ROM of the upper limb, and combined with the needs of ADL, this paper proposes a novel upper limb rehabilitation robot with 6 DOFs (3 active DOFs and 3 passive DOFs) on a wheelchair, as shown in Figure 3. The red revolute joints indicate the active DOFs, which are rotated by the motor. The flexion/extension, adduction/abduction of the shoulder joint and flexion/extension of the elbow joint are controlled by the shoulder joint 1, the shoulder joint 2, and the elbow joint, respectively. The blue revolute joints indicate the passive DOFs, which can be manually rotated. The blue double-headed arrow indicates that the length can be adjusted.

**Figure 4:**
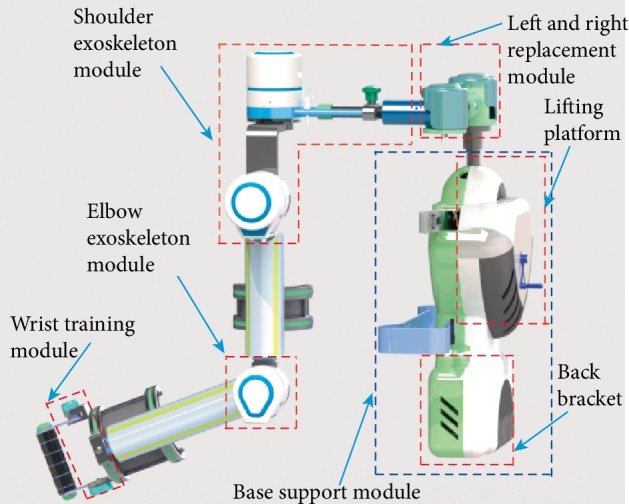
Exoskeleton modules.

This proposed mechanism is designed as an exoskeleton arm that consists of a base support module based on the wheelchair, a right and left replacement module, a shoulder exoskeleton module, an elbow exoskeleton module, and a wrist training module as shown in Figure 4. The exoskeleton is appropriate for the hemiplegic patients who need to sit on wheelchairs.

#### 2.2.2. The Base Support Module and the Right and Left Replacement Module

**Figure 5:**
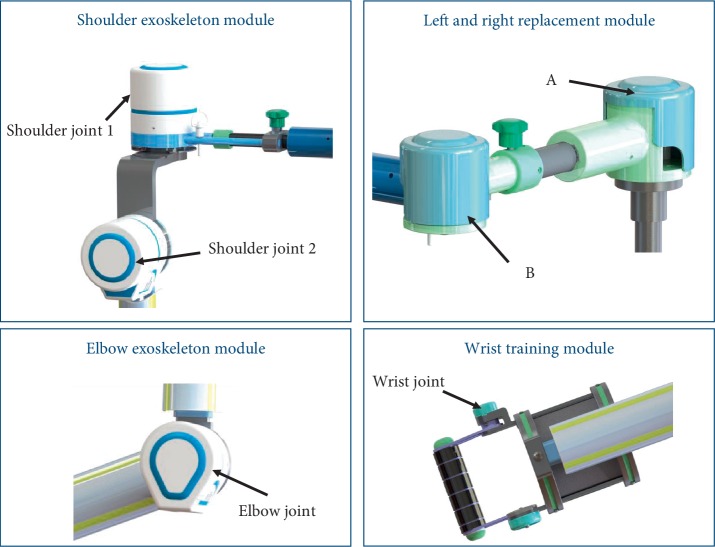
The joints of the exoskeleton.

The base support module has two functions. One is the base of the exoskeleton system named back bracket that mounted on the back of a wheelchair. And the other is to adapt the different heights of patients by lifting platform. The hemiplegic patients have right affected side and left affected side. The right and left replacement module is composed of mechanism A and B shown in Figure 5. The module makes the exoskeleton suitable for hemiplegic patients with different affected sides.

#### 2.2.3. The Shoulder Exoskeleton Module

The shoulder exoskeleton module with 2 DOFs realizes flexion/extension and abduction/adduction. In Figure 5, the abduction/adduction movements are supported by the shoulder joint 1 consisted of one DC motor, harmonic reducer, and one potentiometer. The motion center is designed at the acromioclavicular joint according to the kinesiology of musculoskeletal system functions for rehabilitation [13]. The shoulder joint 2 similar to the shoulder joint 1 is utilized to support the upper arm to do the flexion and extension movements.

#### 2.2.4. The Elbow Exoskeleton Module and the Wrist Training Module

The elbow exoskeleton module realizes flexion/extension of the elbow joint and length adjustment of the forearm. The wrist training module has a passive DOF for flexion/extension of wrist joint to protect the wrist joint of patients and proceed with active training in this design.

#### 2.2.5. Design of Machanical Position Limitation

**Figure 6:**
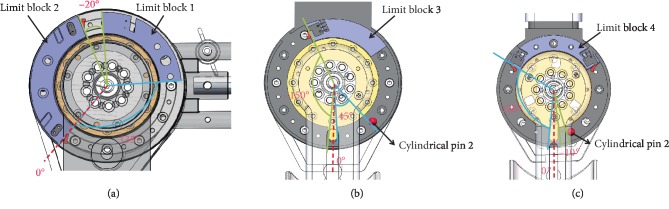
The mechanical limit of the exoskeleton. (a) The mechanical limit of shoulder joint 1. (b) The mechanical limit of shoulder joint 2. (c) The mechanical limit of elbow joint.

To ensure the safety of the exoskeleton on a wheelchair, mechanical position limitation is required for active joints of the exoskeleton. In Figure 6(a), the limit block 1 moves with the shoulder joint 1, and the limit block 2 is fixed. When the shoulder joint 1 moves, the limit block 1 is limited by the limit block 2 between 135 degrees of abduction and 20 degrees of adduction. And the dotted line is the zero position of the shoulder joint 1.

In Figure 6(b), both the cylindrical pin 1 and the limit block 3 are fixed. The shoulder joint 2 is limited by the cylindrical pin 1 and the limit block 3 during moving. And flexion of the shoulder is less than 150° and extension of the shoulder is less than 45°. The dotted line is the zero position of the shoulder joint 2.

In Figure 6(c), both the cylindrical pin 2 and the limit block 4 are fixed. The elbow joint is limited by the cylindrical pin 2 and the limit block 4 during moving. And flexion of the elbow is less than 120° and extension of the elbow is less than 10°. The dotted line is the zero position of the elbow joint.

## 3. Results and Discussion

### 3.1. Kinematics Analysis

**Figure 7:**
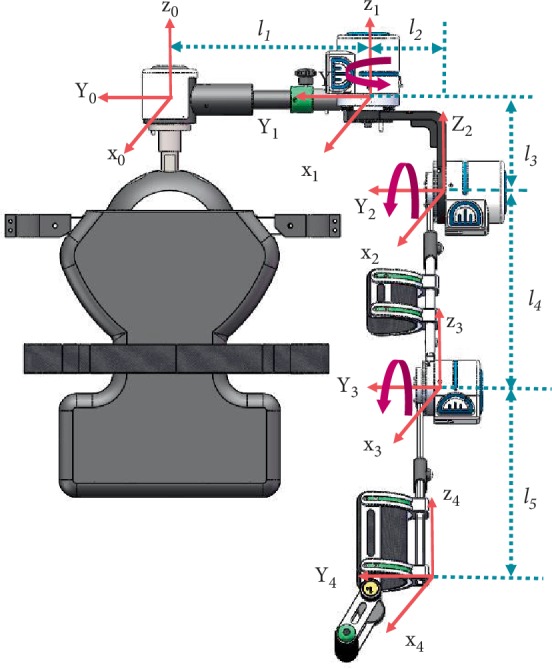
Build coordinate systems for the exoskeleton.

**Figure 8:**
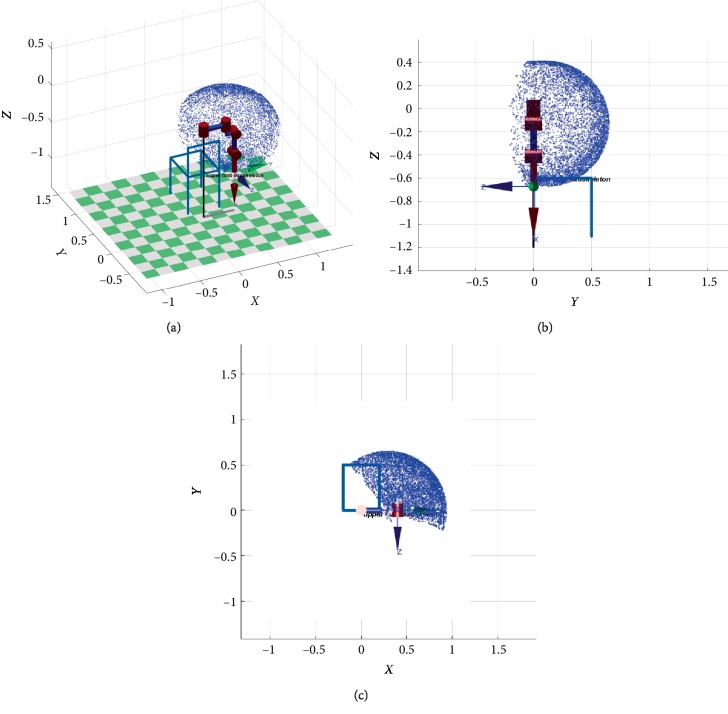
The resulting motion space of the exoskeleton. (a) Three-dimensional view. (b) *Y*–*Z* view. (c) *X*–*Y* view.

To further illustrate the safety of the exoskeleton based on wheelchairs, kinematic analysis of the exoskeleton is performed. Firstly, build the coordinate systems of the exoskeleton in Figure 7.* l_i_* is the length of the exoskeleton. Secondly, calculate kinematic equation of the exoskeleton by coordinate changes, following as Equations (1)–(5). ^*i*^*Ai* + 1 describes the posture and position of a joint. *θ*_1_ is the abduction/adduction of the shoulder. *θ*_2_ is the flexion/extension of the shoulder. *θ*_3_ is the flexion/extension of the elbow. According to the mechanical limit of the exoskeleton mentioned above, the maximum of abduction and adduction of *θ*_1_ are 135° and 20°. The maximum of flexion and extension of *θ*_2_ are 150° and 45°. The maximum of flexion and extension of *θ*_3_ are 120° and 10°. Finally, the resulting motion space of the exoskeleton is shown in Figure 8. The sketch of the chair indicates the position of a wheelchair in space and its lengths are the same as a wheelchair. The blue dots are the trajectory of the shoulder, elbow and wrist joints. Figure 8 shows that the motion of the exoskeleton does not interfere with the chair, which can further determine the safety of the exoskeleton on a wheelchair.

(1) A10=cosθ1sinθ100−sinθ1cosθ10−l100100001,

(2) A21=cosθ20sinθ20010−l2−sinθ20cosθ2−l30001,

(3) A32=cosθ30sinθ300100−sinθ30cosθ3−l40001,

(4) A43=10000100001−l50001,

(5) A40=A10A21A32A43.

(*l*_1_ = 0.287 m, *l*_2_ = 0.112 m, *l*_3_ = 0.130 m, *l*_4_ = 0.291 m, *l*_5_ = 0.260 *m*.)

### 3.2. Smooth Pursuit Movement Test

**Figure 9:**
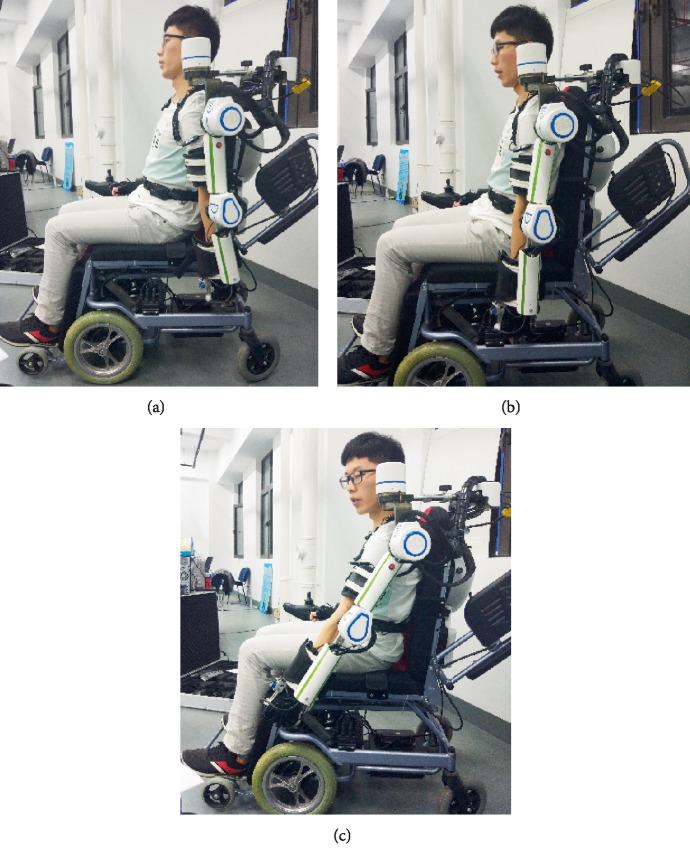
Pursuit movement experiment for shoulder flexion/extension movements.

**Figure 10:**
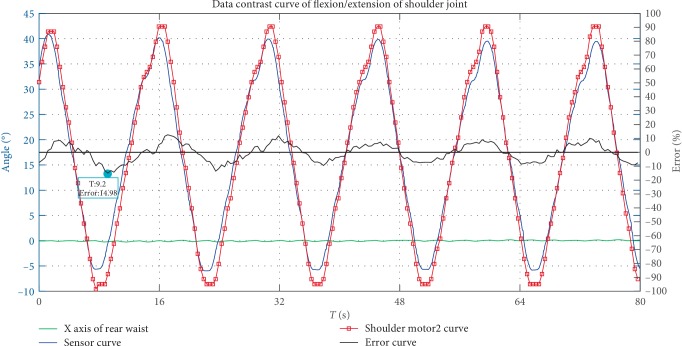
Analysis of the ability of a pursuit movement for flexion/extension of the shoulder joint.

**Figure 11:**
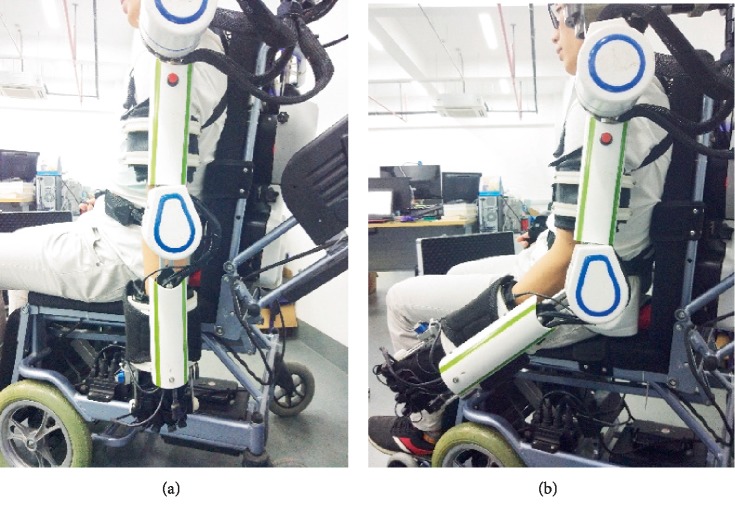
Pursuit movement experiment for elbow flexion/extension movements.

**Figure 12:**
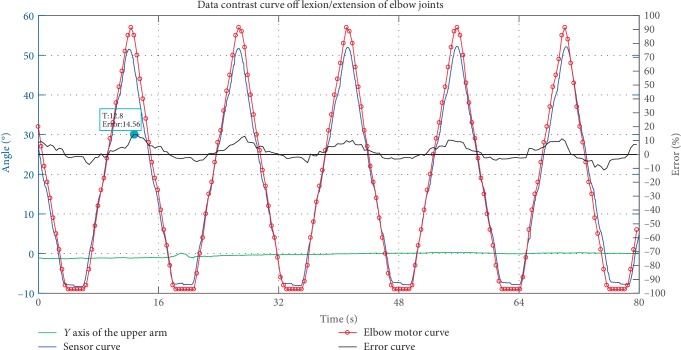
Analysis of the ability of pursuit movements for flexion/extension of elbow joint.

A preliminary prototype was manufactured in order to evaluate the proposed mechanism with 6 DOFs. This paper performed a series of smooth pursuit movement experiments with a healthy male subject who is 29 years old to evaluate the design, effectiveness of the proposed mechanism. Smooth pursuit movement is the movement of the tester's arm driven by the exoskeleton robot. The smooth pursuit motion test is to measure the errors between the joint angles measured by pose sensors of the exoskeleton and the motion analysis system during continuous movement. There were two groups of experiments for testing the smooth pursuit movement characteristics between the passive human arm and the active mobile exoskeleton arm as shown in Figures 9 and 11. These test motions were including the shoulder's flexion/extension, and the elbow's flexion/extension. The motion analysis system was utilized to test the motion angles of the human arm in real time. Three pose sensors were set at the shoulder joint 1, 2 and elbow joint to test the motion angles of the proposed mobile exoskeleton arm. Two groups of angles of motion for each group experiment are obtained and compared together as shown in Figures 10 and 12, respectively.

The motion error analysis between the human arm and exoskeleton arm is given in Equation (6).

(6) $$\begin{array}{c} \delta =\left(\frac{\beta -\gamma }{\beta }\right)\times 100\%, \end{array}$$

where, *δ*-actual relative error, (*γ*-motor movement angle, *β*-sensor angle). The smooth pursuit movement experiment about shoulder flexion and extension movements is shown in Figures 9 and 10. The blue line denotes the real-time angle values obtained by pose sensor at shoulder 2, and the red line with point denotes the real-time angle values obtained by the motion analysis system. The maximum error is less than 15% at *t* = 9.2s, error = 14.98%.

The smooth pursuit movement experiment about elbow flexion and extension movements is shown in Figures 11 and 12. The blue line denotes the real-time angle values obtained by pose sensor at the elbow, and the red line with point denotes the real-time angle values obtained by the motion analysis system. The maximum error is less than 15% at *t* = 12.8s, error = 14.56%.

The motion errors between the human arm and exoskeleton arm are affected by the measurement. The test is a person wearing the motion analysis system that moves under the drive of the exoskeleton. The number of DOFs of the human arm is greater than that of the exoskeleton. The joints' angles of the human arm measured by the motion system are affected by other nontraining degrees of freedom. In addition, the maximum motion errors all occur at the peak of the joints' angles in Figures 10 and 12. The maximum angles of the exoskeleton are always larger than the maximum angles measured by the motion analysis system, the joints angles obtained by the two measurement methods are in the ROM of shoulder and elbow joint on a wheelchair as shown in Table 2. Therefore these errors can be acceptable. Additional passive DOFs for improving the compliance between the exoskeleton and human arm will be considered in the future.

### 3.3. Assisting Drinking Water Test

**Figure 13:**
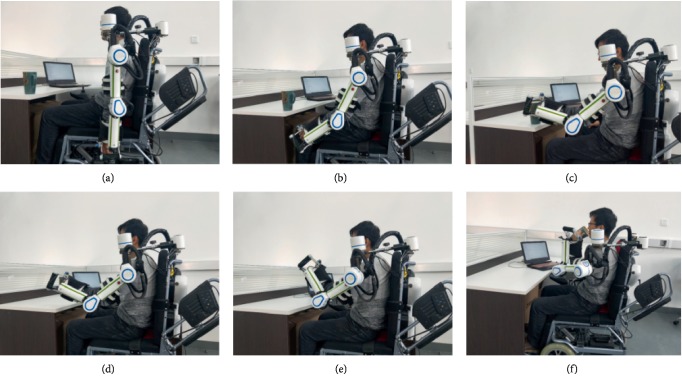
The experiment of drinking water by the exoskeleton.

**Figure 14:**
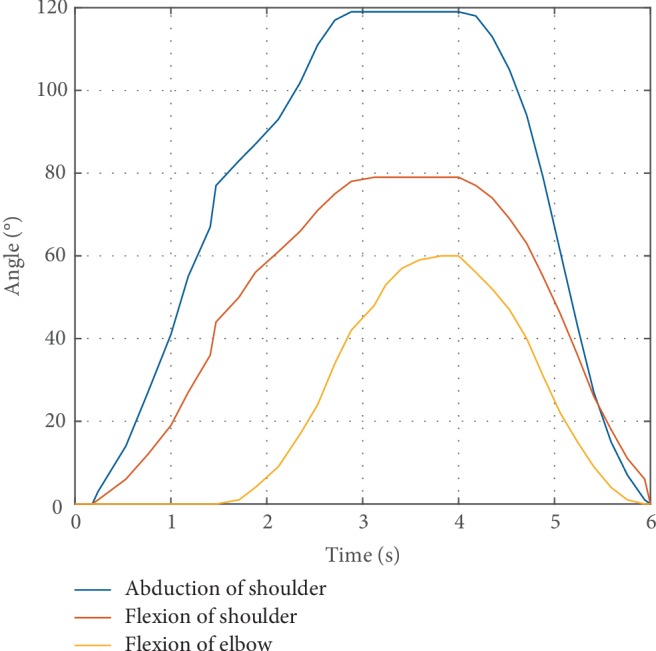
Rotation of exoskeleton joints during drinking.

**Figure 15:**
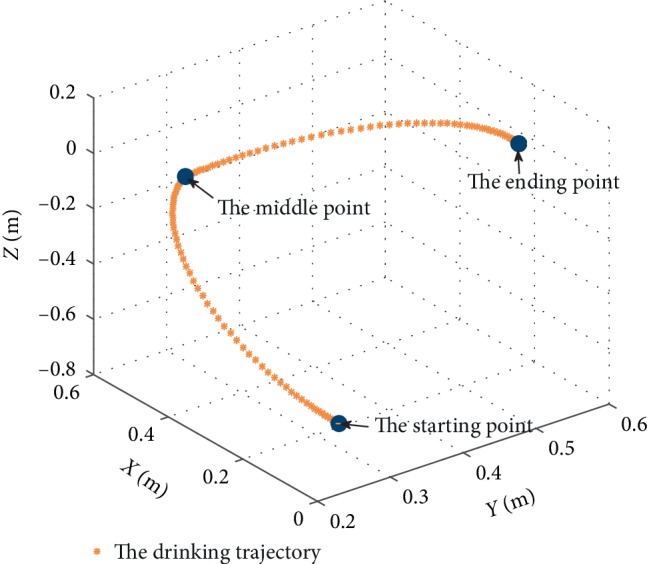
The drinking trajectory of the end-effector of the exoskeleton.

To verify the function compensating ability of the proposed upper limb exoskeleton, the drinking water test was designed. Drinking water was subdivided into two movements. One was taking the cup, the other was raising the cup to the mouth. The test process is shown in Figure 13. It required flexion/extension, abduction/adduction of the shoulder, and flexion/extension of the elbow during drinking water. The results of the upper limb joints' angles in the movement are shown in Figure 14. The red curve is the angles of shoulder adduction. The blue curve is the angle of shoulder flexion. The green curve is the angle of elbow flexion. And the blue dots are discrete points of the joints, angles collected on the control system. The abduction of the shoulder is between 0 and 60 degrees. The flexion of the shoulder is between 0 and 80 degrees. The flexion of the elbow is between 0 and 119 degrees. The experimental results are in the ROM of shoulder and elbow joint on a wheelchair as shown in Table 2. The drinking trajectory of the end-effector of the exoskeleton is shown in Figure 15. The three blue dots in Figure 15 from bottom to top are the starting point, the middle point, and the ending point of the drinking trajectory. The trajectory shows that the motion of the exoskeleton does not interfere with the chair. Therefore, it proves that the exoskeleton robot can assist the patients in daily activities in a wheelchair and it can ensure the safety of using the exoskeleton based on a wheelchair.

### 3.4. Discussion

The aim of this study is to propose a new powered exoskeleton for upper limb rehabilitation based on a wheelchair. The prototype with base support module, shoulder exoskeleton module, elbow exoskeleton module, and wrist training module is designed. The real-time motion trail errors show that the proposed mobile exoskeleton arm can perform the pursuit movements of shoulder and elbow joint well and prevent the second injury of the user. The proposed powered exoskeleton is completely active. The motion of the wearer is driven by the motor directly. Some researches proposed mechanical structure by different actuators. Stienen et al. designed a rotational hydroelastic actuator for a powered exoskeleton for upper limb rehabilitation. The rotational hydroelastic actuator consisted of a rotational hydraulic actuator and a custom-designed symmetric torsion spring in a series-elastic configuration [14]. Hsieh et al. presented a new parallel actuated shoulder exoskeleton that consisted of two spherical mechanisms, two slider crank mechanisms, and a gravity balancing mechanism. Linear series elastic actuators were proposed to obtain accurate force and impedance control at the exoskeleton–limb interface [15]. Qingcong Wu et al. applied bowden-cable actuators with a high power-weight ratio in the upper limb rehabilitation robot system to provide remote power transmission and simplify the mechanical design [16]. It is important for the mechanical design of upper limb exoskeleton to solve the problem of large size and weight. The prototype is controlled totally by the force signal between the upper limb and the mechanical arm. Zhijun Li et al. presented adaptive impedance control of an upper limb robotic exoskeleton using biological signals. The proposed novel impedance algorithm transferred stiffness from a human operator through the surface electromyography signals, being utilized to design the optimal reference impedance model [17]. Peternel et al. proposed an exoskeleton control method for adaptive learning of assistive joint torque profiles in periodic tasks. The human muscle activity was used as feedback to adapt the assistive joint torque behavior in a way that the muscle activity was minimized [18]. The control strategy based on the multi-source signal will be an important research direction.

This work still has some limitations. The power control system and control strategy will be introduced in the next work. The experiments with stroke patients will be discussed in the following research.

## 4. Conclusions

In this paper, a mobile exoskeleton with 6 degrees of freedom (DOFs) based on a wheelchair is proposed according to the range of motion (ROM) training for each joint and activities of daily living (ADL) training for the patients on wheelchairs. The overall proposed system can provide the needed rehabilitation training and compensate for the weak upper-limb function during the wheelchair users' rehabilitation involving task-oriented activities. A prototype of the proposed mobile exoskeleton based on a wheelchair was manufactured and the test of pursuit movement feature was carried out to verify the training safety of the proposed mechanism. The results show that the proposed mobile exoskeleton arm based on wheelchairs designed according to the ROM of ADL with wheelchairs has high motion homogenization. Therefore, patients with wheelchairs can conduct rehabilitation training safely on their wheelchairs by using the proposed system.

## Figures and Tables

**Table 1:** The details of the test.

Age	Sex	Height	Upper arm length	Forearm length	Hand length
29y	Male	172 mm	32 mm	25.1 mm	18.2 mm

**Table 2:** The ROM of the upper limb joints on the wheelchair.

Parameters	Average value (°)	Confidence interval of average value (°)	Standard deviation	Desired value(°)
^*S*^ *θ*_max_^flex^	−150.92	(−152.54, 149.29)	2.12	−150
^*S*^ *θ*_max_^*ext*n^	46.78	(45.77, 47.77)	1.20	45
^*S*^ *θ*_max_^add^	133.25	(131.45, 135.04)	2.51	135
^*S*^ *θ*_max_^abd^	−21.20	(−22.61, −19.78)	1.98	−20
^*S*^ *θ*_max_^int^	−44.85	(−46.27, −43.42)	1.99	−45
^*S*^ *θ*_max_^*ext*l^	72.54	(71.10, 73.98)	2.51	70
^E^ *θ*_max_^flex^	119.31	(118.42, 120.18)	1.23	120
^E^ *θ*_max_^*ext*n^	−9.613	(−10.03, −9.18)	0.55	−10
^F^ *θ*_max_^pron^	82.05	(81.11, 82.98)	1.211	80
^F^ *θ*_max_^supi^	−58.51	(−59.39, −57.62)	1.15	−55
^W^ *θ*_max_^flex^	−49.29	(−49.80, −47.76)	1.64	−45
^W^ *θ*_max_^extn^	54.00	(52.46, 56.53)	1.65	55
^W^ *θ*_max_^ulnar^	13.74	(13.40, 14.06)	0.39	10
^W^ *θ*_max_^radical^	−29.74	(−30.44, −29.03)	0.91	−30

## Data Availability

The reader can get data from the author.
